# Time–frequency texture descriptors of EEG signals for efficient detection of epileptic seizure

**DOI:** 10.1007/s40708-015-0029-8

**Published:** 2016-01-16

**Authors:** Abdulkadir Şengür, Yanhui Guo, Yaman Akbulut

**Affiliations:** 1 Technology Faculty, Electrical and Electronics Engineering Department, Firat University, Elazig, Turkey; 2 Department of Computer Science, University of Illinois at Springfield, Springfield, IL USA

**Keywords:** EEG signal, Time–frequency image, Texture descriptor, Support vector machines, Epileptic seizure detection

## Abstract

Detection of epileptic seizure in electroencephalogram (EEG) signals is a challenging task and requires highly skilled neurophysiologists. Therefore, computer-aided detection helps neurophysiologist in interpreting the EEG. In this paper, texture representation of the time–frequency (*t*–*f*) image-based epileptic seizure detection is proposed. More specifically, we propose texture descriptor-based features to discriminate normal and epileptic seizure in t–f domain. To this end, three popular texture descriptors are employed, namely gray-level co-occurrence matrix (GLCM), texture feature coding method (TFCM), and local binary pattern (LBP). The features that are obtained on the GLCM are contrast, correlation, energy, and homogeneity. Moreover, in the TFCM method, several statistical features are calculated. In addition, for the LBP, the histogram is used as a feature. In the classification stage, a support vector machine classifier is employed. We evaluate our proposal with extensive experiments. According to the evaluated terms, our method produces successful results. 100 % accuracy is obtained with LIBLINEAR. We also compare our method with other published methods and the results show the superiority of our proposed method.

## Introduction

Epileptic seizure is a physiopathological disease that is known as a neurological disorder caused by the transient and unexpected electrical disturbance of the brain. Electroencephalogram (EEG), which is a common method for detection of the epileptic seizure, constructs a representative signal containing information about the brain’s electrical activity. Interpretation of EEG signals for manual detection of the epileptic seizure is not an easy task and requires high skills of neurophysiologists. Moreover, manual interpretation of the long recordings is tedious and time consuming. Therefore, an automated system to help neurophysiologists in detecting epileptic seizures is in great demand. Such an automated system is composed of two main parts [1–4]: EEG feature extraction and classification. While EEG feature extraction enables to characterize EEG signals, classification finds different categories in the input EEG signals.

Detection of epileptic seizures on EEG signals is a popular research topic and many methods have been proposed [2–7]. In these methods, the representative EEG features were extracted either in the time domain [2–4] or frequency domain [8]. The features from time domain are generally extracted from the amplitude or rhythmicity of EEG signals. The frequency domain features are generally computed on the spectrum of EEG signals. There are also several methods based on the time–frequency (*t*–*f*) representation [9–11]. The *t*–*f* image-based features are used to describe the non-stationary nature of the EEG signals. Instantaneous frequency and sub-band energies are other important *t*–*f* domain features for the EEG characterization. In addition, multiscale representations of EEG signals represent rich features. For instance, the statistics of the wavelet coefficients and their relative energies are useful features for EEG classification [6].

Recently, several novel t–f features were proposed based on t–f image descriptors for the automatic detection of epileptic seizure in EEG data. In [10], the authors described visually the normal and epileptic seizure patterns in the *t*–*f* domain. The proposed features are based on Haralick’s texture features calculated from the *t*–*f* representation of EEG signals. In [11], the authors proposed an approach for automatic detection of epileptic seizures using combined Hilbert–Huang transform and support vector machine (SVM) on the *t*–*f* image. Several statistical features such as mean, variance, skewness, and kurtosis of pixel intensity in the histogram of segmented gray-scale *t*–*f* image are considered. Other *t*–*f* image-based features were used to represent the EEG signals in [9]. The authors used a smoothed pseudo Wigner–Ville distribution to obtain the *t*–*f* images. The obtained *t*–*f* images were then segmented on the frequency bands of the EEG signals’ rhythms. These features from the histogram of segmented *t*–*f* images were then used for a multiclass least squares SVM. In [12], the authors combined signal analysis and image processing for classifying EEG abnormalities. The combination of signal-based features and *t*–*f* image-related features was employed to merging key instantaneous frequency descriptors. The proposed method was used to recognize the EEG abnormalities in both adults and newborns.

Our main motivation arises due to the following conclusions:

1. First of all, we think that the *t*–*f* representation of healthy and epileptic seizure EEG signals contain different motifs. Especially, when the frequency bands of the EEG signals’ rhythms are considered, the justification of our motivation becomes more convincing. Because, each rhythm region of the *t*–*f* image for healthy and epileptic seizure has considerably discriminatory texture.
2. These motifs can successfully be modeled by various texture descriptors for further analysis. To this end, texture encoders such as GLCM, TFCM, and LBP are considered to re-shape the *t*–*f* images and a number of statistical quantities are calculated.
3. The considered texture encoders are well known in the image processing and pattern recognition communities with numerous advantageous. These methods are quite efficient in characterizing various texture motifs. Their implementations are easy and complexities are quite low.

In this paper, texture representation of the *t*–*f* image-based epileptic seizure detection is proposed. More specifically, we propose texture descriptor-based features to discriminate normal and epileptic seizure in the *t*–*f* domain. The features that are obtained on the GLCM are contrast, correlation, energy, and homogeneity. Moreover, in TFCM method, the calculated features are mean convergence, code variance, code entropy, uniformity, first-order difference moment, first-order inverse difference moment, second-order difference moment, second-order inverse difference, and four energy distribution values from the co-occurrence matrix. In addition, for the LBP, the histogram is used as the feature. In the classification stage, a support vector machine (SVM) classifier is considered. We evaluate our proposal with extensive experiments. According to the evaluated terms, our method produces successful results. 100 % accuracy is obtained with LIBLINEAR. We also compare our method with other existing methods, and the results show the superiority of our proposal.

In [10], the authors used Haralick’s texture features to classify the healthy and epileptic EEG signals. Our work is different from the previous one such that we search each frequency rhythms and concatenate the features of each rhythm for constructing robust descriptors. Moreover, to the best of our knowledge, TFCM and LBP methods are firstly considered for EEG signal classification in this work and achieved better results in our paper. The rest of the paper is organized as follows: in Sect. 2, the methodology and the related theories are given. In Sect. 3, the experimental works and the obtained results are presented. We conclude the paper in Sect. 4.

## Methodology

**Fig. 1:**
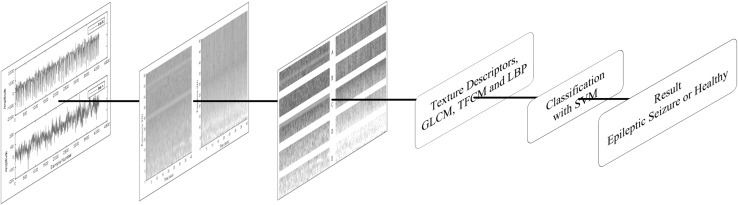
The proposed method

In this work, *t*–*f* representation, texture descriptors, and SVM-based methodology are proposed for the classification of EEG signals as healthy and epileptic seizures. An illustration is given in Fig. 1. As it is observed from Fig. 1, the EEG signals are firstly transformed into *t*–*f* domain. The Spectrogram of Short-Time Fourier Transform (STFT) is used in order to obtain the *t*–*f* images of EEG signals. The obtained *t*–*f* images are then converted into 8-bit gray-scale images and are divided into five sub-images corresponding to the frequency bands of the rhythms. The GLCM, TFCM, and LBP texture descriptors are employed to extract distinctive features for classification purposes. The standard combination of SVM, LIBLINEAR, and Homogenous mapping is investigated for obtaining high-accuracy results in classifying the EEG signals.

### STFT spectrogram

The STFT spectrogram is defined as the normalized, squared magnitude of the STFT coefficients [13]. According to a non-mathematical definition, STFT coefficients can be obtained using a sliding window in time domain in order to divide the signal into small parts and then analyze each part with Fourier transform to determine the frequencies. Thus, a time-varying spectrum can be obtained. In a mathematical view, the STFT can be defined as

$$X(n,\omega ) = \sum\limits_{m = - \infty }^{\infty } {x[m]w[n - m]e^{ - j\omega n} },$$

where $x[m]w[n - m]$ is a short-time part of the input signal *x*[*m*] at time *n*. In addition, a discrete STFT is defined as

$$X(n,k) = X(n,\omega )\left|\right._{{\omega = \frac{2\pi }{N}k}},$$

where *N* shows the number of discrete frequencies. Thus, the spectrogram in logarithmic scale is defined as

$$S(n,k) = \log |X(n,k)|^{2}.$$

### GLCM features

**Fig. 2:**
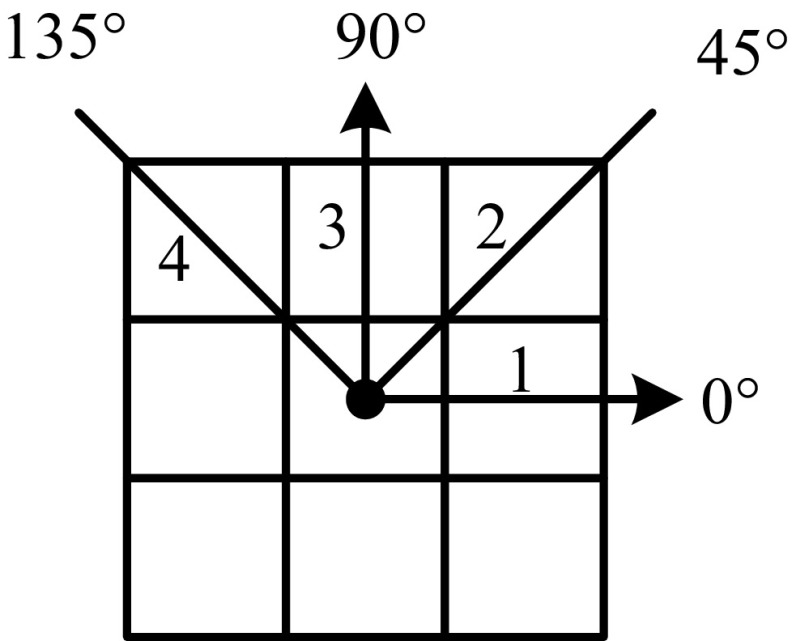
Angular nearest neighbors

GLCM features are commonly used in various image processing applications such as texture segmentation and classification, biomedical image analysis, scene segmentation, etc. [14]. GLCM can be seen as a directional pattern counter with a specific distance *δ* and angle *θ* between neighboring image pixel pairs for gray-scale images. This situation is represented in Fig. 2.

In a numerical view, for *θ* = 0° and *δ* = 1, the GLCM can be defined as

$$M_{\delta ,\theta = 0} (p,q) = \sum\limits_{n = 1}^{N} {\sum\limits_{m = 1}^{K} {\left\{ {\begin{array}{ll} 1 &\quad {{\text{if}}\;I(n,m) = p\,{\text{and}}\,I(n,m + \delta ) = q} \\ 0 &\quad {{\text{otherwise}}} \\ \end{array} } \right.} },$$

where p, q = 0, 1,… *L* – 1; *L* is the number of gray scales; *N* and *K* are the sizes of the image. After normalizing the GLCM, the contrast, correlation, energy, and homogeneity features are calculated.

### TFCM features

**Fig. 3:**
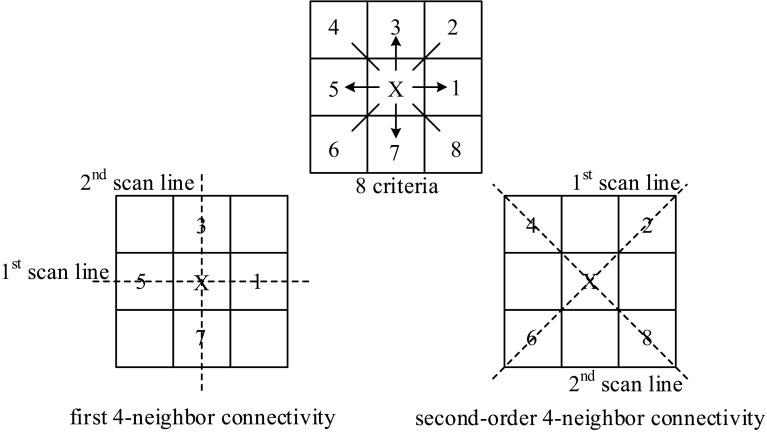
Horizontal, vertical, and diagonal connectivity sets

The TFCM translates a gray-scale input image into a texture feature number image via differencing in the image domain followed by successive stages of vector classification [15]. The algorithm firstly calculates the differences along horizontal, vertical, and diagonal connectivity sets. Figure 3 shows the related illustrations.

The resulting two-element difference vectors are thresholded at a tolerance into quantized two-element vectors whose values are from the set of {−1, 0, 1}, interpreted as negative, no change, and positive difference, respectively. The TFCM maps the individual quantized difference vectors to gray-level class numbers based on the degree of the variation in each vector [15]. Then a mapping procedure is employed for further coding gray-level class numbers. The following mapping is further employed for obtaining final 2-D texture feature number images. After constructing the co-occurrence matrices of texture feature number images, 12-dimensional feature vector is calculated [15].

### LBP features

**Fig. 4:**
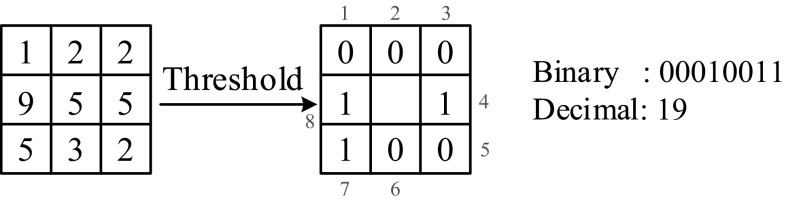
LBP procedure after the LBP image is constructed; the histogram of the LBP image is used as the feature

Ojala et al. developed an operator called LBP for describing the local textural patterns [16]. This simple but effective operator has been then used as a texture descriptor in many image processing-based applications. The LBP works in a 3 × 3 pixel block and the pixels in this block are thresholded by its center pixel value, multiplied by powers of two and then summed to obtain a label for the center pixel. Figure 4 shows the basic idea of the LBP operator. The center pixel’s gray-scale value becomes 19 after applying the LBP procedure. The mathematical illustration of the procedure is as follows:

$$LBP(x) = \sum\limits_{i = 1}^{8} {f(G(x_{i} ) - G(x))2^{i - 1} }$$

$$f(t) = \left\{ {\begin{array}{ll} {1,} &\quad {t \ge 0} \\ {0,} &\quad {t < 0} \\ \end{array} } \right.,$$

where *x* shows the location of the center pixel, *x*_*i*_ shows the ith neighboring pixel as shown in Fig. 4, and *G*(.) is the gray-scale value of a pixel.

## Experimental work

**Fig. 5:**
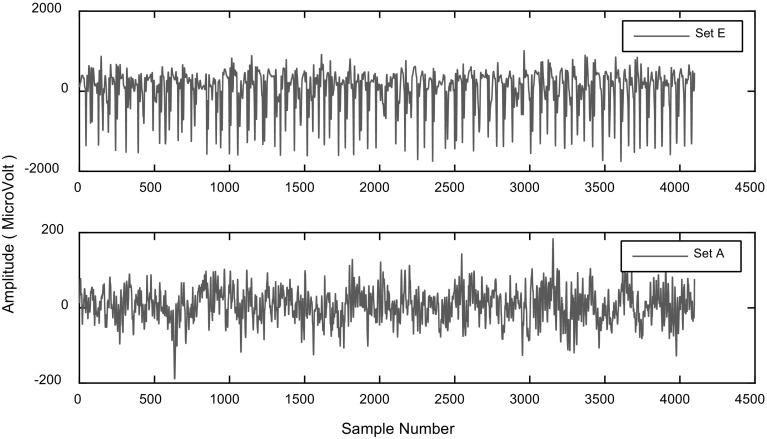
Illustration of EEG signals, Set E and Set A

The experiments are conducted on an open source EEG dataset that was recorded in Bonn University [17]. The recorded dataset has five sets denoted as A to E. Each contains 100 single-channel EEG signals, and each one having 4097 samples. In other words, each recorded EEG signal has 23.6 s duration. The datasets A and E are considered. While set A was taken from surface EEG recordings of five healthy volunteers with eyes open and closed, respectively, set E only contains epileptic seizure. Figure 5 shows a typical EEG illustration of both healthy and epileptic seizure. As shown in Fig. 5, the amplitudes of the epileptic EEG signals are higher than those of the normal EEG signals.

**Fig. 6:**
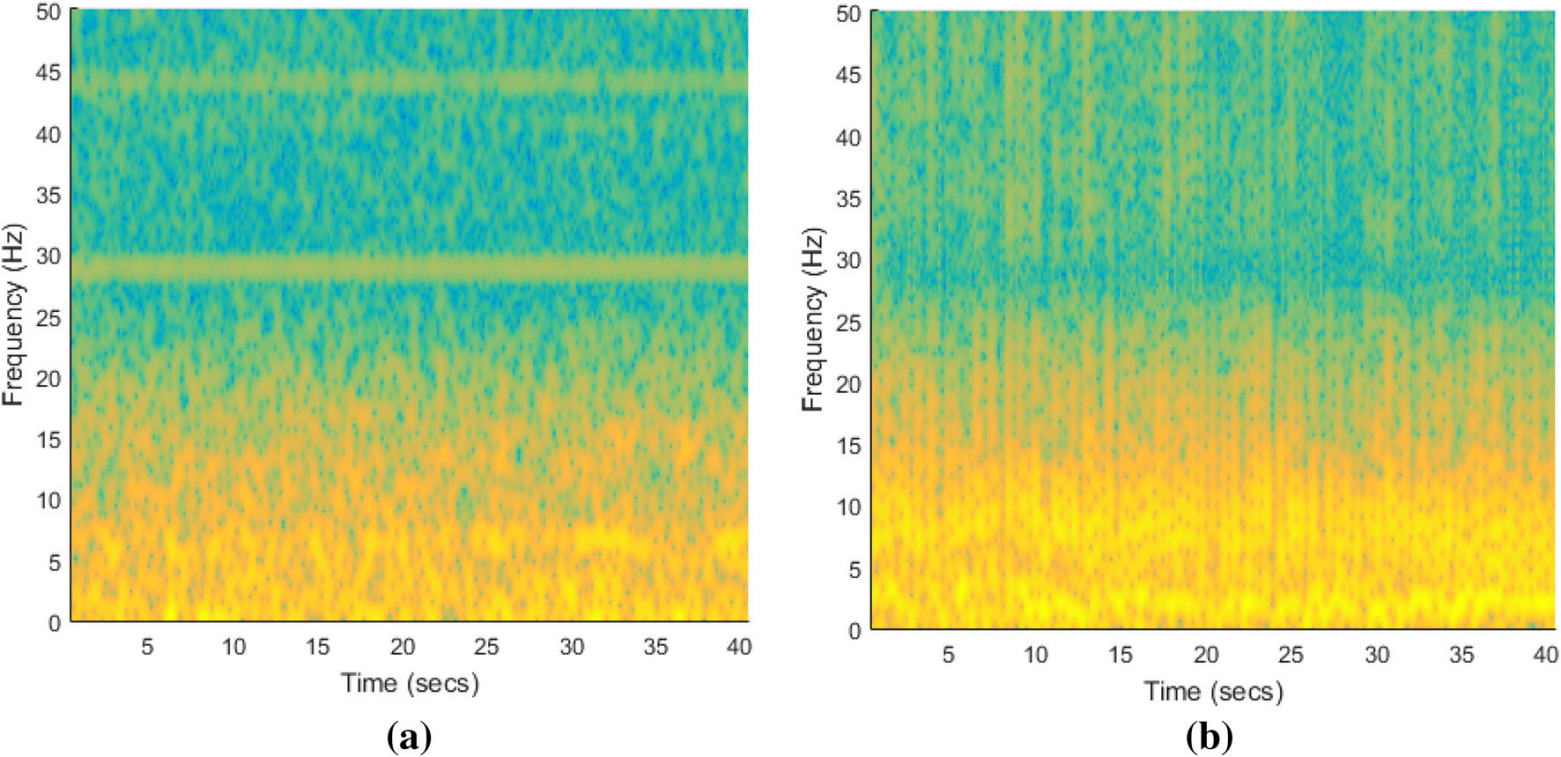
Spectrogram of EEG signal: **a** healthy and **b** epileptic seizure

Moreover, Fig. 6 shows the spectrogram of EEG signals for healthy and epileptic seizure, respectively. By visual inspection, a qualitative discrimination of healthy and epileptic seizure can be seen in Fig. 6. For further processing the t–f images, we convert them into 8-bit gray-scale images. The 8-bit gray-scale t–f images are then divided into five sub-images corresponding to the frequency bands of the rhythms to localize significant structures. The main EEG rhythm on frequency ranges is as follows [9]:

- Delta: 0–4 Hz.
- Theta: 4–8 Hz.
- Alpha: 8–12 Hz.
- Beta: 12–30 Hz.
- Gamma: 30–50 Hz.

**Fig. 7:**
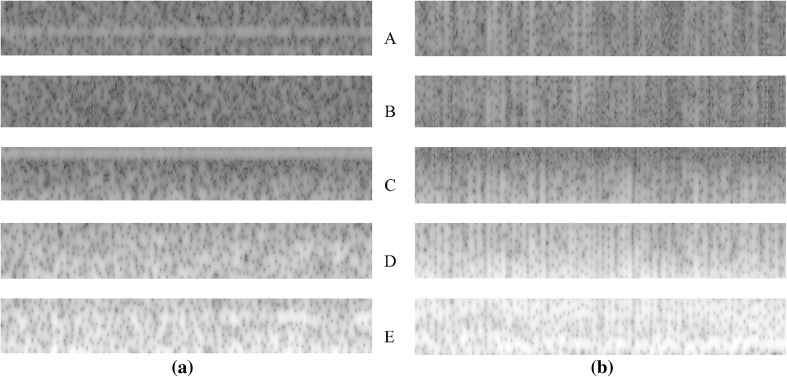
Gray-scale sub-images: **a** healthy and **b** epileptic seizure EEG signal. *A* gamma, *B* beta, *C* alpha, *D* theta, *E* delta

In Fig. 7, we show the divided sub-images corresponding to frequency bands of the rhythms.

After gray-scale sub-images (Fig. 7) for healthy and epileptic seizure EEG signal are constructed, the texture descriptors are computed. For computing the GLCM, the distance parameter is set to 1 and the angle parameter value ranges from 0^o^ to 135^o^ with a 45^o^ increment. Thus, 4 GLCMs are obtained and by calculating the contrast, correlation, energy, and homogeneity features, a 16-dimensional feature vector is constructed for each sub-image. Moreover, for obtaining the TFCM features, once each sub-image is converted to a texture feature number, a 12-element feature vector is generated based on these co-occurrence matrices and texture feature number histograms. The tolerance parameter of the TFCM is set to 80. In addition, for LBP, the histogram is computed for each sub-images. Thus, a 256-dimensional feature vector is obtained. Finally, feature vectors that are extracted from each sub-image are concatenated. In this case, three of 80-, 60-, and 1280-dimensional feature vectors are constructed for the GLCM, TFCM, and LBP, respectively.

The linear SVM is employed in the classification stage of our proposal [18]. Moreover, the homogeneous mapping is considered to increase the efficiency of the SVM [20, 21]. This mapping procedure enables a compact linear representation of the input dataset. Thus, a very fast linear SVM classifier can be obtained. The VLFeat tool is used for both homogeneous mapping and FV encoding [19]. The VLFeat open source library implements various computer vision algorithms such as Fisher Vector, VLAD, SIFT, MSER, SLIC superpixels, large-scale SVM training, and many others specializing in image understanding and local feature extraction and matching. We also use the LIBLINEAR for further increasing the efficiency of the SVM [20, 21]. LIBLINEAR was developed as an open source library for large-scale linear classification.

To evaluate the performance of the proposed scheme, we employ classification accuracy, sensitivity, and specificity.

$${\text{Sensitivity}}\;{ = }\;\frac{\text{TP}}{{{\text{TP}}\; + \;{\text{FN}}}}$$

$${\text{Specificity}}\;{ = }\;\frac{\text{TN}}{{{\text{TN}}\; + \;{\text{FP}}}}$$

$${\text{Accuracy}}\; = \;\frac{{{\text{TP}}\;{ + }\;{\text{TN}}}}{{{\text{TP}}\;{ + }\;{\text{TN}}\;{ + }\;{\text{FP}}\;{ + }\;{\text{FN}}}},$$

where TP represents the total number of correctly detected true-positive samples and TN represents the number of correctly detected true-negative samples; FP and FN represent the total number of false-positive and false-negative samples, respectively.

The setup parameters of the classifiers are adjusted for obtaining the best performance. For the SVM, we experiment with all kernels and the best result is obtained with a linear kernel. The *C* parameter is set to 100. L_2_-regularized L_2_-loss solver is chosen for LIBLINEAR. In addition, the *C* parameter for LIBLINEAR is set to 0.07. Chi^2^ kernel is used for homogeneous mapping. It is worth mentioning that the experimental results are recorded using fivefold cross-validation. The overall performance of the proposed method is tabulated in Table 1.

**Table 1:** Obtained results of GLCM features

Classifier structure	Accuracy (%)	Sensitivity (%)	Specificity (%)
SVM	92.5	95	90
LIBLINEAR	99.5	100	99
Homogenous Mapping + LIBLINEAR	100	100	100

The results suggest that the best accuracy is obtained with Homogenous Mapping + LIBLINEAR. The classification accuracy is 100 %. In addition, the sensitivity and specificity values are 100 and 100 %, respectively. The SVM yields the worst classification results. 92.5 % accuracy is recorded. Sensitivity and specificity values are 95 and 90 %, respectively. Thus, it is obvious that LIBLINEAR structure greatly improves the performance. LIBLINEAR structure is 7 % better than that of SVM. In addition, homogeneous mapping also improves performance. 0.5 % more accurate result is obtained with homogenous mapping than LIBLINEAR structure.

Similar experiments are carried out for TFCM features. The related classifier parameters are set as the follows: the SVM kernel is chosen as a polynomial and *C* is set to 1. L1-regularized L2-loss solver is chosen for LIBLINEAR. In addition, the *C* parameter for LIBLINEAR is set to 15.

The performance results of TFCM features are shown in Table 2. The best accuracy is obtained using Homogenous Mapping + LIBLINEAR. The obtained accuracy is 87 %. The LIBLINEAR and SVM obtain the same classification accuracy. 82 % is tabulated. The other sensitivity and specificity values can be seen in Table 2. The best sensitivity value is obtained with Homogenous Mapping + LIBLINEAR. The worst specificity value is recorded for SVM (79 %).

**Table 2:** Obtained results of TFCM features

Classifier structure	Accuracy (%)	Sensitivity (%)	Specificity (%)
SVM	82	85	79
LIBLINEAR	82	80	84
Homogenous Mapping + LIBLINEAR	87	90	84

We conclude our experiments with the LBP features. We adjust the related parameters of the classifiers for obtaining high-performance results. Similar to previous experiments, the intersection kernel is chosen for the SVM. We also experiment with other kernels such as linear, radial basis function, polynomial, and sigmoid. The intersection kernel achieves the highest accuracy. The *C* parameter is selected as 0.32. L1-regularized L2-loss solver is chosen for LIBLINEAR. In addition, the *C* parameter for LIBLINEAR is set to 100.

The performance results of LBP features are shown in Table 3. The best accuracy is obtained using SVM and LIBLINEAR. The obtained accuracy is 100 % for both classification methods. Actually, this is a surprising result because in the previous two experiments, the Homogenous Mapping + LIBLINEAR structure yields better results than SVM and LIBLINEAR. Homogenous Mapping + LIBLINEAR yields the worst results for LBP features.

**Table 3:** Obtained results of LBP features

Classifier structure	Accuracy (%)	Sensitivity (%)	Specificity (%)
SVM	100	100	100
LIBLINEAR	100	100	100
Homogenous Mapping + LIBLINEAR	99.5	100	99

We also compare our results with other published methods handling the classification problem in the same dataset A and E. The results are shown in Table 4. From Table 4, we can see that accuracy of our proposed method is higher compared with other methods.

**Table 4:** Obtained results of LBP features

Researchers	Method	Accuracy (%)
Polat et al. [22]	FFT, decision tree	98.72
Subasi [6]	DWT, mixture of expert model.	95
Fu et al. [11]	Hilbert–Huang Transform, SVM	99.125
Wang et al. [23]	WT, Entropy, k-NN	99-100
This paper	LBP, SVM, LIBLINEAR	**100**
This paper	GLCM, HM, LIBLINEAR	**100**

The bold numbers show the highest accuracies

## Conclusions

In this work, *t*–*f* representation of EEG signals, texture descriptors, and SVM approach has been used to detect the epileptic seizure. The STFT spectrogram has been considered for discrimination of the epileptic seizure and healthy EEG signals. The obtained *t*–*f* images are then divided on the frequency bands of the rhythms. The features are obtained by calculating the histogram of LBP and various statistical features of the GLCM and TFCM for each *t*–*f* sub-image. The features are then fed into the classifier. The extensive experiments indicate that the LBP features obtained the best results. The second best results are recorded with GLCM features, and finally TFCM-based features exhibit the worst performance. This situation may be caused because of the dimensionality of the feature vectors. In other words, LBP-based feature vector has the higher dimensionality and TFCM-based feature vector has the lowest. In addition, LBP may better characterize the EEG t–f images than the GLCM and TFCM methods.
